# Can back-wall cystic figures of thyroid nodules predict benignity?

**DOI:** 10.1530/ETJ-25-0243

**Published:** 2026-01-09

**Authors:** Jean-Guillaume Marchand, Marie Bienvenu-Perrard, Agnes Rouxel, Cécile Ghander, Camille Buffet, Gilles Russ

**Affiliations:** ^1^ Thyroid and Endocrine Tumors Department, La Pitié-Salpêtrière Hospital, Sorbonne University, GRC No. 16, Paris, France; ^2^ Centre of Pathology and Radiology, Paris, France; ^3^ Nuclear Medicine Department, Cochin Hospital, Paris, France; ^4^ Nuclear Medicine Department, Avicenne Hospital, Bobigny, France

**Keywords:** back-wall cystic figure, thyroid nodule, ultrasound, EU-TIRADS, hyperechoic foci

## Abstract

**Background:**

According to the European Thyroid Association (ETA) 2023 consensus, hyperechoic spots correspond to different subtypes: comet tail artifacts, back-wall cystic figures (BWCF), and microcalcifications. BWCF are supposed to be in favor of benignity. However, this is not well supported by the existing literature.

**Objective:**

To assess whether EU-TIRADS 4 nodules with BWCF have a lower risk of malignancy than those without BWCF. The secondary objective was to compare this risk to that of EU-TIRADS 3 nodules and evaluate indeterminate and benign cytology rates across the three groups.

**Methods:**

Four physicians prospectively included 150 thyroid nodules before fine-needle aspiration: 50 EU-TIRADS 4 with BWCF, 50 EU-TIRADS 4 without BWCF, and 50 EU-TIRADS 3.

**Results:**

The difference in malignancy risk between EU-TIRADS 4 without BWCF and EU-TIRADS 4 with BWCF was significant (*P* < 0.001). EU-TIRADS 3 and EU-TIRADS 4 nodules with BWCF were significantly correlated with Bethesda II cytology: 94 and 82%, respectively, compared to 26% in EU-TIRADS 4 nodules without BWCF (*P* < 0.001). Bethesda III–IV cytology was more frequent in EU-TIRADS 4 nodules without BWCF (62%) than in EU-TIRADS 4 with BWCF (18%) or EU-TIRADS 3 (6%) (*P* < 0.001).

**Conclusion:**

BWCF in EU-TIRADS 4 nodules indicate a low malignancy risk, close to that of EU-TIRADS 3 nodules. These results underline the need to distinguish BWCF from other hyperechoic foci, such as microcalcifications, to improve risk stratification. Recognizing BWCF may allow the use of higher FNA thresholds and should be considered in future guidelines as I-TIRADS.

## Introduction

Thyroid nodules are common in the general population and increase in frequency with age. The vast majority of these nodules are benign (1). The high frequency of benign thyroid nodules has encouraged the development of risk stratification systems to limit the number of recurrent examinations, fine-needle aspirations (FNA), and inappropriate surgeries. The semiology of ultrasound features for risk stratification of malignancy has been refined over time (2). Among these features, intranodular hyperechoic spots (peri-millimeter hyperechoic foci) are often described as microcalcifications. However, according to the European Thyroid Association (ETA) 2023 consensus (1), hyperechoic spots correspond to different subtypes: comet tail artifacts, back-wall cystic figures (BWCF), and microcalcifications. Unlike microcalcifications, BWCF tend to be an argument in favor of benignity. These hyperechoic spots, different from microcalcifications, have been known about for several years. In 2011 Russ *et al.* described pseudo-microcalcifications, which they defined as small, often linear, hyperechoic punctuations located at the back of microcystic cavities (3). Subsequently, in 2017, the ETA issued Guidelines for Ultrasound Malignancy Risk Stratification of Thyroid Nodules in Adults. The EU-TIRADS defined different types of hyperechoic spots, including posterior acoustic enhancement of the back wall of a microcystic area corresponding to linear rather than round hyperechoic spots located at the back portion of microcystic cavities (4). These images were described as rather suggestive of benignity. In the same year, the American College of Radiology (ACR) TI-RADS (5) reported small echogenic foci that probably represent the back walls of tiny cysts (not suspicious), but only in spongiform nodules.

On the other hand, the recent I-TIRADS lexicon (6) details four echogenic foci/calcifications: punctate echogenic foci/microcalcifications (≤1 mm), macrocalcifications (>1 mm), peripheral (rim) calcifications, and echogenic foci with comet-tail artifacts, but no mention is made of BWCF. To our knowledge no study has specifically been published on this subject. Their recognition could lead to a reduction in the number of fine-needle aspirations, especially in EU-TIRADS 4 nodules, which frequently produce indeterminate cytology results (7).

The primary objective of our work was to assess whether the risk of malignancy of EU-TIRADS 4 nodules with BWCF was lower than that of a group of EU-TIRADS 4 nodules without BWCF.

The secondary objectives were, first, to determine if the risk of these EU-TIRADS 4 nodules with BWCF was close to the low risk of malignancy attributed to EU-TIRADS 3 nodules. Second, it was to understand if the proportion of indeterminate cytology results was different between the three groups (EU-TIRADS 3, EU-TIRADS 4 with BWCF and EU-TIRADS 4 without BWCF).

## Materials and methods

### Compliance with ethical standards

This study was conducted in accordance with the principles of the Declaration of Helsinki. This human study was approved by the CERIM Institutional Review Board (CRM-2406-414). Due to the descriptive nature of this study, informed consent was waived.

### Study population

**Figure 1:**
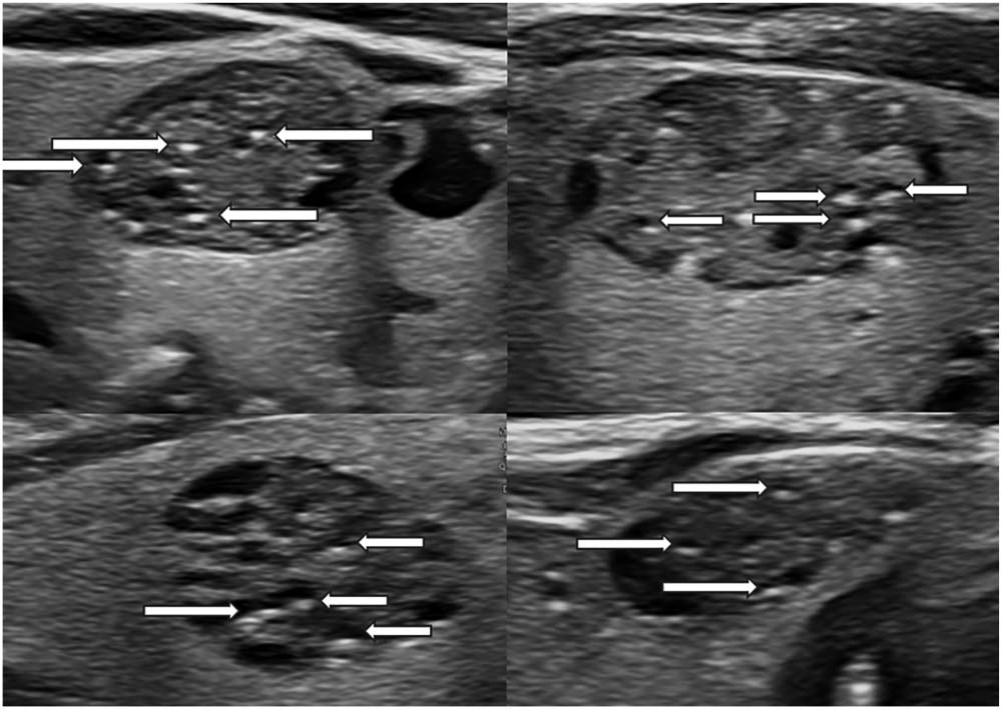
EU-TIRADS 4 nodule with BWCF: linear hyperechoic punctations located behind a microcystic anechogenic cavity (arrow).

**Figure 2:**
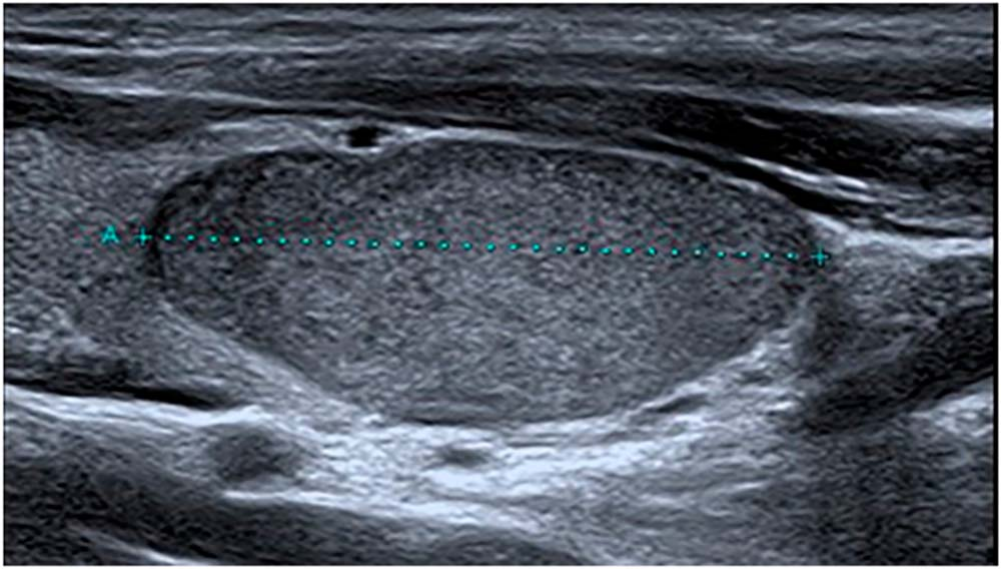
EU-TIRADS 4 nodule without BWCF.

The study was conducted in a high-volume specialized referral center dedicated to thyroid imaging diagnosis and interventional procedures. It included all patients aged 18 and older who were referred to our center for fine-needle aspiration (FNA). Between October 1 and December 20, 2024 we prospectively included the first 50 EU-TIRADS 3 nodules, the first 50 EU-TIRADS 4 nodules with BWCF (Fig. 1), and the first 50 EU-TIRADS 4 nodules without BWCF (Fig. 2).

Exclusion criteria were age under 18, nodules with EU-TIRADS scores 2 and 5, non-diagnostic cytological results Bethesda I, and thyroid stimulating hormone (TSH) < 0.4 unless a scintigraphy showed that the nodule was cold. This criterion aimed to avoid including hyperfunctioning nodules, which are typically associated with a very low risk of malignancy and are not routinely assessed by FNA.

### US examinations and image analysis

All US examinations were performed with a 5–18 MHz (i18LX5) transducer (Aplio i700, Canon Medical Systems, USA) equipped with the Prism module. The US features of thyroid nodules were prospectively evaluated before US-guided biopsy by four physicians (GR, AR, MBP, and JGM) specialized in diagnostic and interventional ultrasound imaging of the thyroid and neck, with more than 10 years of experience in the field. Conventional gray-scale ultrasound was used.

All thyroid nodules were scored using the EU-TIRADS score (4).

To assess the presence of BWCF, the definition of the lexicon from the ETA 2023 guidelines supplementary material was used (1). BWCF are defined as linear hyperechoic punctations located behind a microcystic anechogenic cavity (Fig. 1). This definition must be strictly applied; in particular, visualization of the microcystic cavity, otherwise they more likely are hyperechoic punctations of undetermined type, as defined in the 2017 ETA guidelines (4). To enhance the visualization of the hyperechoic foci, zooming on the nodule, raising the US emitting frequency, and reducing the dynamic range and compound imaging treatment was systematically applied. This, in turn, helps to differentiate from true microcalcifications, which correspond to round hyperechoic foci located in the solid part of the nodule. Among BWCF, two subgroups were considered according to the number of hyperechoic foci: less than three and three or more, to determine if their number had an impact on malignancy risk.

### Thyroid FNA and cytological interpretation

FNA was performed for all nodules using a capillary action US-guided FNA technique and 27 G needles. One or two passes were made per nodule for cytological analysis, and all results were reported according to the 2023 Bethesda system (8).

To determine if the risk of malignancy of EU-TIRADS 4 nodules with BWCF was lower than that of a group of EU-TIRADS 4 nodules without BWCF, Bethesda V and VI results were considered malignant, while Bethesda II was considered benign. The proportion of these two groups was compared between EU-TIRADS 4 nodules with BWCF and without BWCF.

To assess if the risk of EU-TIRADS 4 nodules with BWCF was close to the low risk of malignancy attributed to EU-TIRADS 3 nodules, the percentage of Bethesda II results was compared between these two groups.

Finally, to determine if the proportion of indeterminate cytology results was different between the three groups, Bethesda III and IV results were considered indeterminate, and the proportion of these was compared between EU-TIRADS 4 nodules with BWCF and without BWCF and EU-TIRADS 3 nodules.

### Statistical analysis

Summary descriptive statistics of qualitative data were expressed as numbers and percentages, and those of quantitative data as means and standard deviations. Statistical analyses responding to primary and secondary objectives used the standard Fisher’s exact test in the case of two-by-two classification tables. The generalized Fisher’s exact test was used in the case of greater tables, and the nonparametric Kruskal–Wallis test for the comparison of means. Because few patients were included for two nodules, the observations may not have been completely independent. This may violate the independence assumption required by statistical tests and marginally affect the reliability of the results. After exclusion of multiple observations per patient, a confirmatory analysis was conducted on the restricted sample containing a single nodule per patient. A *P*-value lower than 0.05 was considered significant. All analyses used SAS software for Windows, release 9.4.

## Results

Of the 137 patients included in the study, 31 (22.7%) were males and 106 (77.3%) were females. In these patients, 150 thyroid nodules were included, divided into three groups: 50 EU-TIRADS 4 with BWCF, 50 EU-TIRADS 4 without BWCF, and 50 EU-TIRADS 3. The mean nodule size (±SD) was 26 mm ± 12.1 mm. TSH values were available for a subset of patients in each group (*n* = 9–11), with a mean overall TSH of 1.5 ± 1.0 mIU/L. There was no significant difference in TSH levels between the three groups (Kruskal–Wallis test, *P* = 0.23). Data are summarized in Table 1.

**Table 1:** Thyroid nodule and population characteristics.

	*n*	EU-TIRADS 3	EU-TIRADS 4	
			With BWCF	Without BWCF
Mean nodule size (mm)	50	29.5 ± 8.9	20.8 ± 9.4	27.6 ± 15.2
Mean age (years)	50	57.9 ± 12.3	59.9 ± 13.1	56.0 ± 14.9
Mean TSH (mUI/L)	9	2.1 ± 1.3	1.3 ± 1.5*	1.3 ± 0.8

* *n* = 11.

Cytology results according to TI-RADS score and presence of BWCF are summarized in Table 2. No cases of Bethesda V–VI cytology were observed in the EU-TIRADS 3 group (0%) or in the EU-TIRADS 4 group with BWCF (0%). In contrast, six cases (12%) of Bethesda 5–6 cytology were identified in the EU-TIRADS 4 group without BWCF. Statistical analysis using Fisher’s exact test showed that the difference in malignancy risk between EU-TIRADS 4 without BWCF and each of the two other groups (EU-TIRADS 3 and EU-TIRADS 4 with BWCF) was significant (*P* < 0.001 for both comparisons). These differences were confirmed (*P* < 0.001) in the restricted database retaining one single nodule per patient (44 EU-TIRADS 3, 46 EU-TIRADS 4 with BWCF, and 47 EU-TIRADS 4 without BWCF). BWCF in EU-TIRADS 4 nodules showed an estimated sensitivity of 100% (54–100%) and a specificity of 53% (43–64%).

**Table 2:** Cytology results by EU-TIRADS category and BWCF presence, and statistical comparisons.

Cytological category	EU-TIRADS 3 (1)	EU-TIRADS 4		*P* values		
		With BWCF (2)	Without BWCF (3)	(1) vs (2)	(2) vs (3)	(1) vs (3)
Bethesda				0.11	<0.001	<0.001
VI	0/50 (0%)	0/50 (0%)	3/50 (6%)			
V	0/50 (0%)	0/50 (0%)	3/50 (6%)			
IV	0/50 (0%)	4/50 (8%)	9/50 (18%)			
III	3/50 (6%)	5/50 (10%)	22/50 (44%)			
II	47/50 (94%)	41/50 (82%)	13/50 (26%)			
Malignant						
Bethesda V–VI	0/50 (0%)	0/50 (0%)	6/50 (12%)		0.027	0.027
95% CI			4.5–24.3			
Indeterminate						
Bethesda III–IV	3/50 (6%)	9/50 (18%)	31/50 (62%)	0.12	≤0.001	≤0.001
95% CI	1.2–16.6	8.6–31.5	47.2–75.4			
Benign						
Bethesda II	47/50 (94%)	41/50 (82%)	13/50 (26%)	0.12	<0.001	<0.001
95% CI	83.5–98.8	68.6–91.4	14.6–40.3			

Benign cytology (Bethesda II) was observed in 94% of EU-TIRADS 3 nodules and 82% of EU-TIRADS 4 nodules with BWCF, compared to only 26% in EU-TIRADS 4 nodules without BWCF. The difference in proportions across the three groups was statistically significant (generalized Fisher’s exact test, *P* < 0.001). Pairwise comparisons revealed a highly significant difference between EU-TIRADS 4 without BWCF and both EU-TIRADS 4 with BWCF (*P* < 0.001) and EU-TIRADS 3 (*P* < 0.001). The difference between EU-TIRADS 3 and EU-TIRADS 4 with BWCF did not reach statistical significance (*P* = 0.12).

The proportion of indeterminate cytological results (Bethesda categories III and IV) was evaluated in the three groups. Among EU-TIRADS 3 nodules, 6% had indeterminate cytology. In the EU-TIRADS 4 with BWCF group, this proportion increased to 18%. Notably, the highest proportion was observed in the EU-TIRADS 4 without BWCF group, with 62% showing Bethesda III or IV cytology. The generalized Fisher’s exact test showed a highly significant difference in indeterminate cytology rates among these three groups (*P* ≤ 0.001). Pairwise comparisons indicated significant differences between EU-TIRADS 4 without BWCF and EU-TIRADS 4 with BWCF (*P* ≤ 0.001), and between EU-TIRADS 4 without BWCF and EU-TIRADS 3 (*P* ≤ 0.001). However, no significant difference was found between EU-TIRADS 3 and EU-TIRADS 4 with BWCF (*P* = 0.12). These findings suggest that the absence of BWCF in EU-TIRADS 4 nodules is strongly associated with a higher likelihood of indeterminate cytological results.

The relationship between the number of BWCF per nodule and the risk of malignancy was also explored. Although the number of cases was limited, among the seven nodules with fewer than three punctate echogenic foci, five were classified as Bethesda II and two as Bethesda III. In comparison, among the 43 nodules with three or more punctate echogenic foci, 36 were Bethesda II, 3 were Bethesda III, and 4 were Bethesda IV. The difference in the distribution of cytological categories according to the number of BWCF was not statistically significant (Fisher’s exact test, *P* = 0.22).

## Discussion

This study highlights three key findings regarding the diagnostic value of BWCF in EU-TIRADS 4 nodules. First, the presence of BWCF was associated with a significantly lower risk of malignancy compared to nodules without BWCF. Second, EU-TIRADS 4 nodules with BWCF showed a proportion of Bethesda II cytology results similar to EU-TIRADS 3 nodules, suggesting a comparable malignancy risk. Third, the absence of BWCF was strongly associated with a higher rate of indeterminate cytological results. These findings suggest that the presence or absence of BWCF can help define two distinct risk subgroups within EU-TIRADS 4 nodules, potentially refining the indications for FNA.

To our knowledge, this study is the first to assess the impact of BWCF on the risk of malignancy in EU-TIRADS 4 nodules. These nodules represent a heterogeneous group with a 6–17% risk of malignancy (4) and approximately 24% of all thyroid nodules (9). Most studies of hyperechoic spots have focused on microcalcifications, which are strongly associated with malignancy. They are defined as round echogenic foci around 1 mm in size without posterior shadowing, located in the solid component of a nodule (4, 10). Some older studies have investigated different types of these echogenic foci, but the risk of malignancy and the diagnostic value of different types of echogenic foci have not been fully established (11, 12). Moreover, in these reports, the subcategory BWCF is never mentioned. In a more recent study from 2023, only hyperechoic punctuations <1 mm in the solid part of the nodule without comet-tail artifact increased the risk (13). This corresponds to the ETA definition of microcalcifications, but again, there was no mention of BWCF. One possible explanation is that many of these studies were conducted using older ultrasound equipment. To enhance detection, older-generation systems required specific manual settings such as one focal point, magnification, switching off the compound, and reducing the dynamic range. However, many current-generation ultrasound machines are equipped with high-frequency probes (18 MHz) and automated signal processing, making the detection of BWCF feasible and reliable with no modification of usual settings in routine practice.

Our results suggest that the absence of BWCF in EU-TIRADS 4 nodules significantly increases the risk of malignancy compared with EU-TIRADS 3 and EU-TIRADS 4 nodules with BWCF. Furthermore, the proportion of Bethesda II cytology results is similar between EU-TIRADS 3 and EU-TIRADS 4 with BWCF. These nodules are likely to bear a malignancy risk close to that of EU-TIRADS 3 nodules. One possible explanation is that cystic features (whether microcystic areas, partially cystic nodules, or minimal cystic changes) are generally associated with benignity, as suggested by Na *et al.*’s study on minimal cystic changes in thyroid nodules (14).

These observations support the notion that EU-TIRADS 4 nodules with BWCF may represent a distinct, lower-risk subgroup. Given their lower malignancy risk similar to EU-TIRADS 3, a more conservative approach to their management may be considered. However, our study was not designed to assess specific size thresholds for FNA. Further prospective studies are needed to evaluate whether current FNA recommendations should be adjusted for this subgroup.

In our study, we also observed a significantly higher percentage of indeterminate cytological results in EU-TIRADS 4 nodules without BWCF (62%) compared to those with BWCF (18%). Few studies have examined the correlation between TI-RADS score and Bethesda classifications. In the study by Bozer *et al.*, 66.9% of TI-RADS 4 nodules were classified as Bethesda III (15), while Vargas-Uricoechea *et al.* reported that Bethesda III and IV accounted for 58% of TI-RADS 4 nodules (16). Although these studies did not account for BWCF status, our findings raise the hypothesis that the proportion of indeterminate cytology in EU-TIRADS 4 nodules may be higher when EU-TIRADS 4 with BWCF are excluded. This suggests that molecular testing could be particularly relevant in this subgroup.

This study has several limitations. It was conducted at a single center with a relatively small sample size. In addition, histological confirmation was not available. However, this reflects routine clinical practice, where benign cytological results (Bethesda II), the predominant category in our cohort, are generally managed without surgery. Restricting inclusion to surgically resected nodules would have introduced a selection bias toward higher-risk cases and would not have aligned with the primary objective of evaluating ultrasound features in a general population of thyroid nodules.

These findings provide a foundation for future multicenter studies with larger cohorts to validate and build upon our results.

## Conclusion

This study is the first to demonstrate that the presence of back-wall cystic figures (BWCF) in EU-TIRADS 4 thyroid nodules is associated with a significantly lower risk of malignancy compared to EU-TIRADS 4 nodules without BWCF, close to that of EU-TIRADS 3 nodules. These findings highlight the importance of distinguishing BWCF from other hyperechoic foci, such as microcalcifications, to refine risk stratification within the EU-TIRADS 4 score. The absence of BWCF in EU-TIRADS 4 nodules was also strongly correlated with a higher rate of indeterminate cytological results. By recognizing BWCF as a marker of benignity, clinicians could reduce unnecessary FNAs, particularly for EU-TIRADS 4 nodules with BWCF, potentially setting a higher FNA threshold. In addition, future guidelines on US risk stratification of thyroid nodules, especially the I-TIRADS, could incorporate this feature (6).

## Declaration of interest

The authors declare that there is no conflict of interest that could be perceived as prejudicing the impartiality of the work reported.

## Funding

This research did not receive any specific grant from any funding agency in the public, commercial, or not-for-profit sector.

## Author contribution statement

JGM and GR designed the current study. All authors created and/or managed the original databases. JGM and GR wrote the initial manuscript. All authors reviewed and revised the manuscript to improve its intellectual and technical content.
